# Megakaryopoiesis and Platelet Biology: Roles of Transcription Factors and Emerging Clinical Implications

**DOI:** 10.3390/ijms22179615

**Published:** 2021-09-05

**Authors:** Ji-Yoon Noh

**Affiliations:** Immunotherapy Research Center, Korea Research Institute of Bioscience and Biotechnology (KRIBB), 125 Gwahak-ro, Yuseong-gu, Daejeon 34141, Korea; nohj16@kribb.re.kr

**Keywords:** megakaryocyte, platelet, transcription factor, differentiation, disease, ageing

## Abstract

Platelets play a critical role in hemostasis and thrombus formation. Platelets are small, anucleate, and short-lived blood cells that are produced by the large, polyploid, and hematopoietic stem cell (HSC)-derived megakaryocytes in bone marrow. Approximately 3000 platelets are released from one megakaryocyte, and thus, it is important to understand the physiologically relevant mechanism of development of mature megakaryocytes. Many genes, including several key transcription factors, have been shown to be crucial for platelet biogenesis. Mutations in these genes can perturb megakaryopoiesis or thrombopoiesis, resulting in thrombocytopenia. Metabolic changes owing to inflammation, ageing, or diseases such as cancer, in which platelets play crucial roles in disease development, can also affect platelet biogenesis. In this review, I describe the characteristics of platelets and megakaryocytes in terms of their differentiation processes. The role of several critical transcription factors have been discussed to better understand the changes in platelet biogenesis that occur during disease or ageing.

## 1. Introduction

Platelets are small, anucleate blood cells produced from mature bone marrow megakaryocytes [1]. In humans, the reference range for normal platelet count is 150–400 × 10^9^/L, and its life span is 8–12 days. Approximately 10^11^ platelets are generated daily, suggesting that a robust differentiation process is required [2]. Thrombocytopenia, a condition characterized by defective or low platelet generation, can be life-threatening owing to the risk of hemorrhage. Thus, many studies have been conducted to understand the mechanisms of thrombopoiesis, and novel factors involved in its mechanism are being discovered. Hematopoietic stem cells (HSCs) give rise to megakaryocyte–erythroid progenitors (MEPs) via common myeloid progenitors (CMPs), followed by differentiation into megakaryocytes (MKs) [3]. In addition, recent studies on mice suggest the existence of an MK-biased HSC population, which has been shown to play a critical role in thrombopoiesis during pathologic conditions [4,5,6]. Congenital platelet disorders occur due to quantitative or qualitative defects during megakaryocyte differentiation. Moreover, myeloproliferative neoplasm (MPN), a hematologic malignancy, is characterized by MK hyperplasia due to skewed MK differentiation in the bone marrow, resulting in an abnormal platelet count. Notably, many studies on inherited thrombocytopenia have revealed the accumulation of megakaryocyte precursors (MkPs) and/or early MKs in the bone marrow; this indicates the developmental arrest could result in MkP hyperplasia and affect platelet biogenesis. In this review, I focus on the molecular mechanisms of megakaryopoiesis in order to understand platelet disorders.

## 2. Platelet Biology and Platelet Biogenesis

### 2.1. Platelet Receptors

Although platelets are small and enucleated, they express various types of receptors, such as integrins, glycoproteins, selectins, G-protein coupled receptors (GPCRs), and receptors of the immunoglobulin type. These receptors interact with their endogenous ligands, which include agonists for the initiation of hemostasis, to enforce platelet activation. The receptor–ligand interactions are exemplified by integrin α5β1–fibrinogen [7], integrin α6β1–laminin [7], integrin α2β1–collagen [8], GP1bα–von Willebrand factor (VWF) [9], glycoprotein VI (GPVI)–collagen [10], GPIIb/IIIa (integrin α_2b_β_3_, CD41/CD61)–fibrinogen [11], and p-selectin–p-selectin glycoprotein ligand1 (PSGL1) [12]. Platelets adhere to VWF on the subendothelial matrix via the GPIb/V/IX complex when vascular damage occurs [13]. This adhesion is stabilized by the exposed collagen, which recruits the binding of GPVI and integrin α2β1 to the platelets.

The heterodimeric integrin GPIIb/IIIa, which binds to fibrinogen, VWF, and fibronectin, comprises up to 15% by weight of total platelet plasma membrane proteins [14]. On initiation of GPVI signaling after vascular injury, crosslinking of platelets with platelets and platelets with endothelial cells occur via the interaction between GPIIb/IIIa and fibrinogen, leading to thrombus formation [15,16]. During hemostasis and thrombosis, agonists such as ADP, thromboxane A2 (TxA_2_), and thrombin, which are secreted by activated platelets or synthesized through the coagulation cascade in the plasma membrane, are released and stimulate the platelets further via P2Y purinoceptor 12 (P2RY_12_), TxA_2_ receptor, and protease activated receptor 1 (PAR1), respectively [17,18,19]. This supports platelet aggregation and thrombus formation [20]. Inhibitory signaling pathways, induced by nitric oxide (NO) and prostacyclin (PGI2), to avoid undesired platelet activation also exist [21].

**Figure 1:**
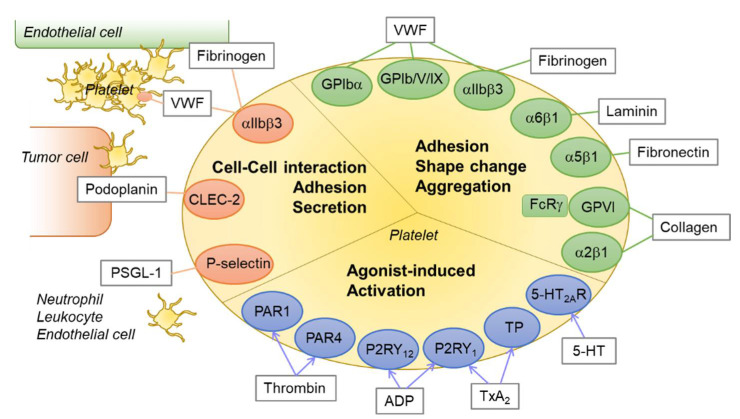
An overview of platelet receptor–ligand interactions. Platelets express various receptors for agonists or integrins and glycoproteins for ligands. The receptor–ligand interactions play critical roles in platelet activation, aggregation, shape change, adhesion, and thrombus formation. Green circle: integrins and glycoproteins for platelet adhesion and aggregation; blue circle: platelet receptors for agonists; orange circle: integrins and lectins for platelet–cell interaction; rectangle with gray line: endogenous ligands or agonists for each receptor. Abbreviations: PAR1, protease activated receptor 1; P2RY12, P2Y purinoceptor 12; TP, thromboxane receptor; 5-HT2AR, 5-hydroxytryptamine (serotonin) receptor 2A; CLEC-2, C-type lectin-like receptor 2; VWF, von Willebrand factor; PSGL-1, P-selectin glycoprotein ligand-1.

Selectins are involved in the aggregation of platelets with other cell types, including endothelial cells, neutrophils, and circulating tumor cells [22,23]. Although it has been reported that tumor cell-induced platelet aggregation (TCIPA) or cancer-associated thrombocytosis is prevalent, its mechanism is not fully understood. Platelet p-selectin (CD62P), which is stored in platelet alpha granules, is exposed at the cell surface upon platelet activation via degranulation. A variety of p-selectin ligands, such as CEA, CA125, MUC1, and CD44 variants, are expressed in cancer cells [24,25]. In addition to the receptors for hemostasis, platelet CLEC-2 has also been identified as a receptor for podoplanin in tumor cells [26,27]. Podoplanin has been shown to mediate downstream signaling of CLEC-2 through an immunoreceptor tyrosine-based activation motif (ITAM), which modulates kinases for platelet activation [28]. Platelet GPIIb/IIIa also mediates platelet–tumor cell binding under shear stress [29,30]. Taken together, these results show that platelets could assist in tumor metastasis by crosslinking the circulating tumor cells, and thus, targeting the platelet receptors has been suggested as a potential anticancer therapy [31,32,33,34]. An overview of platelet receptor–ligand interactions and their roles is depicted in Figure 1.

### 2.2. Platelet Metabolism

Platelet lifespan, which is approximately 8–12 days, likely depends on the mitochondria. The packaging of 5–8 mitochondria per platelet is critical for their survival and proper function. Mitochondria are not only involved in adenosine triphosphate (ATP) production [35], but also in reactive oxygen species (ROS) generation, calcium ion homeostasis, and cell processes and viability regulation [36,37]. Platelets are highly metabolically flexible, which enables the cells to function under various conditions [38]. Normally, glycolysis provides approximately 60% of the total ATP in platelets, whereas oxidative phosphorylation (OXPHOS), which occurs in mitochondria, compensates for the remaining 40% [39].

Disease or ageing could induce mitochondrial dysfunction, and are correlated with a decline in platelet survival and a higher risk of thrombosis [40]. For instance, enhanced production of mitochondrial ROS and activation of platelets, presumably due to hyperglycemia, have been observed in diabetes mellitus [41]. Mitochondrial damage and pathologic platelet activities have also been observed in other age-related diseases, such as Parkinson’s [42] and cardiovascular diseases [43], suggesting that platelet mitochondria may serve as therapeutic targets. In addition, higher methylation of platelet mitochondrial DNA (mtDNA) has been suggested as a possible biomarker for cardiovascular disease, as epigenetic regulation of platelet mtDNA could affect platelet activity [43]. Ageing is another factor that determines mitochondrial health [44]. Recently, TNFα has been implicated in increased mitochondrial mass and activity in megakaryocytes, resulting in thrombosis associated with ageing [45]. The alterations in platelet biogenesis and function with ageing will be discussed further.

### 2.3. Platelet Biogenesis

In 1906, MKs in the bone marrow were first identified as the precursors of platelets [46]. These cells are characterized by a large cell size (50–100 μm in diameter), multinuclei (64N DNA in a human cell), and consist of granules, mitochondria, and a demarcation membrane system (DMS). Thrombopoietin (TPO) plays a key role in megakaryopoiesis from hematopoietic stem cells by binding to its receptor myeloproliferative leukemia protein (c-MPL) in the MKs [46,47,48]. Receptor dimerization occurs by TPO binding, resulting in the activation of Janus kinase 2 (JAK2) and signal transducers and activators of transcription (STATs) that promote the maturation of MKs. The TPO-MPL signaling pathway also recruits phosphoinositide-3-kinase (PI3K) and mitogen-activated protein kinases (MAPKs) such as AKT [49]. Knockout of MPL resulted in the reduction of approximately 85% of the platelets, while the remaining 15% may be dependent on other mechanisms.

Mitochondria play an important role in megakaryocyte maturation as they are critical for platelet health. Many studies have demonstrated the involvement of mitochondrial fusion/fission dynamics in energy production, cell division, differentiation, and apoptosis [50,51]. Mitochondrial fission is mediated by dynamin-related protein 1 (DRP1), a cytosolic guanosine triphosphatase (GTPase), which localizes to the mitochondrial outer membrane [52]. This process can be induced by cellular stress, such as an increase in ROS, and is followed by mitochondrial fragmentation [53,54]. Aberrant mitochondrial shape may also lead to enhanced ROS formation, although the correlation between mitochondrial shape and redox homeostasis remains to be elucidated. Mitochondrial fusion is executed by mitofusin 1 and 2 (MFN1 and MFN2, respectively) and optic atrophy 1 (OPA1), and its importance in determining memory T-cell formation has been demonstrated [55,56,57]. Endomitosis is one of the most critical processes of megakaryopoiesis, followed by polyploidization. Thus, it is evident that mitochondrial dynamics and concomitant ROS play an important role in MK function, which is platelet production [58]. Recently, it was shown that mitochondrial ROS initiated proplatelet formation and terminal maturation of MKs [59,60]. In addition, MK deformation was found to be associated with mitochondrial ROS levels, and therefore, proplatelet formation [59], which is consistent with the findings of previous studies in which cytoskeletal reorganization was influenced by ROS [61].

Development of the DMS, which later forms the platelet membrane, is another feature of megakaryocyte maturation [62,63]. Since it is thought that one MK releases more than 3000 platelets, it is critical that the DMS divides the cytoplasm efficiently. The exact mechanism by which this unique membrane system is established is unclear. Hereditary diseases with loss-of-function mutations on *GP1BA*, *GP1BB*, or *GP9*, and *MHC9*-related disorders exhibit an abnormal DMS, resulting in thrombocytopenia [64,65]. Notably, DMS membranes were found to be enriched with phosphatidylinositol 4,5-bisphosphate (PI-4,5-P_2_), which is mediated by PI-5-P-4-kinase α (PIP4Kα) [66]. Knockdown of PIP4Kα resulted in impaired DMS formation with a reduction in the size of the MKs. The authors indicated that PI-4,5-P_2_ might be involved in the development of DMS, in association with the assembly of actin fibers.

## 3. Transcription Factors in Megakaryopoiesis

**Figure 2:**
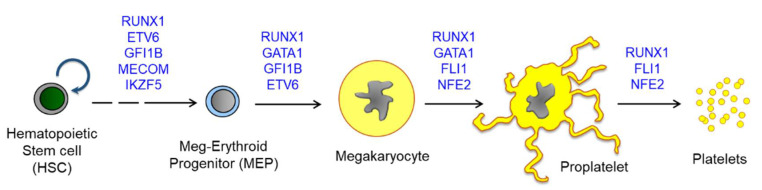
A scheme for megakaryopoiesis to show the involvement of transcription factors in a stepwise manner. Mutations on the transcription factors induce defective megakaryocyte maturation, resulting in congenital thrombocytopenia. Most except for MECOM are found to be associated with abnormal hyperplasia of MkPs or MKs upon mutation. HSCs produce all lineages of hematopoietic cells, and MEPs are differentiated into both megakaryocytes and erythroid cells. Megakaryocytes are further maturated to proplatelets and release platelets into blood flow. One megakaryocyte is known to produce more than 3000 platelets and approximately 10^11^ platelets are generated daily. Abbreviations: RUNX1, Runt-related transcription factor 1; FLI1, friend leukemia virus integration 1; NFE2, Nuclear factor erythroid 2; MECOM, MDS1 and EVI1 complex locus; ETV6, ETS variant 6.

By investigating congenital thrombocytopenia, more than 30 genes have been implicated in megakaryopoiesis and platelet biogenesis [67]. Among them, seven genes, including *RUNX1, GATA1, FLI1, GFI1B, MECOM, ETV6*, and *NFE2,* are transcription factors [68,69,70]. During megakaryopoiesis, transcription factors are activated in a stepwise manner, and thus, different mutations induce different disease characteristics. For instance, *GATA1* mutations could affect both megakaryopoiesis and erythropoiesis [71], whereas variants of *RUNX1* and *ETV6* are involved in leukemia predisposition [72,73]. Other genes such as *HOXA11* and *MECOM* are associated with bone marrow failure [74,75]. Although defects in platelet production induced by these mutations have been investigated, it is also important to examine the alterations occurring in hematopoietic progenitor cells (HPCs) and subsequent megakaryopoiesis, as these are not fully understood. In this section, I describe the genomic landscape of megakaryopoiesis by examining the platelet disorders mediated by mutations in transcription factors. A scheme for megakaryopoiesis and key transcription factors that are associated with platelet biogenesis and platelet disorders are summarized in Figure 2 and Table 1.

### 3.1. RUNX1

Runt-related transcription factor 1 (RUNX1; AML1) plays a critical role in the emergence of all definitive hematopoiesis and represents a common mutational target in leukemia. RUNX1 contributes to hematopoietic progenitor proliferation and is involved in the later stages of MK maturation. In addition, RUNX1, together with its cofactor core-binding factor beta (CBFβ), stimulates various platelet genes, such as platelet factor 4 (*PF4*) [97], nuclear receptor subfamily 4 group A member 3 (*NR4A3*) [98], protein kinase c theta (*PRKCQ*) [99], and myosin light chain 9 (*MYL9*) [76]. It can also generate a transcriptional repressor complex, which is inhibited by the arginine methyltransferase PRMT1, at megakaryocytic promoters [100]. Germline heterozygous mutations in *RUNX1* lead to autosomal dominant human syndrome familial platelet disorder with a predisposition to myeloid malignancy (FPDMM), which presents with thrombocytopenia and bleeding [101]. The MKs of the patients with platelet disorders expressed nonmuscle myosin IIb (MYH10), which was supposed to be downregulated by RUNX1 during endomitosis, a hallmark of megakaryocyte differentiation [77,102,103]. Thus, RUNX1 is involved in polyploidization and proplatelet formation of megakaryocytes by regulating the expression of MYH9, MYL9, and MYH10.

Recently, single-cell RNA-sequencing (scRNA-SEQ) data revealed that haploinsufficiency of RUNX1 in induced pluripotent stem cell (iPSC)-derived MKs resulted in transcriptional deregulation, associated with various immune and cytokine response pathways, including TGF-β1 signaling [78]. The RUNX1 mutant iPSC-derived HPCs showed elevated c-Jun N-terminal kinase (JNK)-2 phosphorylation, a TGF-β1 signaling pathway. Small molecules that inhibited JNK or TGFβR-1 corrected MK yield, which was suppressed by the RUNX1 haploinsufficiency. Notably, growing evidence suggests that MK-biased hematopoietic stem and progenitor cells (HSPCs) display self-renewal and platelet production in response to stress and inflammation [5,104,105]. Therefore, RUNX1 may play an important role in these processes, together with other hematopoietic transcription factors [106].

### 3.2. GATA1

GATA1 is crucial for erythroid–megakaryocytic differentiation, and interacts with its cofactor ZFPM1 (FOG1; friend of GATA1) [107]. A lack of GATA1 function in the MK lineage leads to the accumulation of immature MKs with decreased polyploidization and cytoplasmic maturation, due to decreased cyclin D1 expression [108,109]. Mutations in *GATA1* are known to cause several human disorders [110]. Missense mutations of *GATA1* on FOG1-interacting sequences or nuclear binding sites also affect MK differentiation, resulting in aberrant numbers and maturation of MKs and platelets [111]. In addition, GATA1 interacts with RUNX1 in the zinc finger domain [112]. Down syndrome acute megakaryoblastic leukemia (DS-AMKL) is associated with somatic mutations in *GATA1*. Mutations in exon 2 result in an increase in the short form of GATA1 (GATA1s) protein via alternative splicing of the full-length GATA1 transcript. Many reports have revealed that GATA1s promotes the expansion of MK progenitors, but does not allow their terminal maturation, resulting in pre-leukemic transient myeloproliferative disorder (TMD) [79,80,113,114]. It is thought that the presence of GATA1s expression, trisomy 21, and additional hits, such as overexpression of the ETS family transcription factor ERG, could lead to the development of DS-AMKL [115].

Interestingly, reduced GATA1 levels is represented by the accumulation of MEP/MkPs in the bone marrow or proliferation of the precursors in ex vivo cell culture, in both mice and humans [81,116]. This has led to the development of the *Gata1*-deficient MEP-like cell line G1ME, from murine embryonic stem cells (ESCs), which have been widely used to understand the molecular mechanism of meg–erythroid differentiation, via perturbation of GATA1 expression during hematopoietic differentiation of stem cells [117]. However, G1ME cells failed to undergo terminal differentiation, presumably because GATA1 restoration via retroviral transduction is nonphysiological. This led to the development of G1ME2 cells using an inducible gene knockdown system, which greatly improved the previous system [118]. This indicates that the physiological levels of GATA1 are critical for meg–erythroid differentiation. Reduced *Gata1* expression could lead to the emergence of leukemia and bone marrow fibrosis with the accumulation of immature MKs [119,120]. Although it has not been investigated whether GATA1 levels are correlated with MkP expansion and differentiation in human cells, many studies on GATA1s have demonstrated that the physiological regulation of GATA1 is crucial for these processes.

### 3.3. FLI1

Friend leukemia virus integration 1 (FLI1) increases the expression of megakaryocyte-specific genes, including *MPL*, *ITGA2B* (i.e., CD41), *PF4*, and *GP9* (i.e., CD42a), together with that of GATA1, FOG1, and ETS1, in the late MkPs [121,122]. Several factors are involved in driving the MEPs toward either the erythroid or megakaryocytic lineage [2]. For instance, MYB is a proto-oncogene that induces the expression of the erythroid transcription factor, *KLF1* [123]. It has been demonstrated that the knockdown of *MYB* enhances megakaryocyte differentiation and platelet generation, indicating its role in the inhibition of megakaryopoiesis [124]. The expression of miR-150-5p has been suggested as a mechanism of action to reduce MYB levels during megakaryopoiesis, following TPO-MPL signaling activation [125,126]. In addition, the downregulation of *MYB* induces *FLI1* expression.

Paris–Trousseau syndrome (PTS), an inherited disorder associated with 11q chromosome deletion, is characterized by thrombocytopenia, with an increased risk of bleeding. It occurs owing to the hemizygous deletion of a region that encodes *FLI1* and *ETS1*. It is also characterized by dysmegakaryopoiesis in the bone marrow and giant fused α granules in platelets [82,127]. Both PTS-specific (11q23.3 deletion) and genome-edited (*FLI1* deletion, *FLI1*^+/-^) human iPSCs showed a decrease in the yield of megakaryocytes and platelets, indicating that the FLI1 is crucial for megakaryopoiesis [83]. It has also been discovered that the transcription factor *ETS1,* which is negatively regulated by *FLI1,* is critical in megakaryopoiesis [128]. Furthermore, during the later stages of megakaryopoiesis, the RUNX1/FLI1 complex suppresses ankyrin repeat domain 26 (*ANKRD26*), resulting in the development of blood platelets [129]. Point mutations in the 5′UTR of *ANKRD26* have shown to result in familial thrombocytopenia 2 and leukemia predisposition, mainly mediated by the derepression of *ANKRD26* due to insufficient RUNX1/FLI1 complex binding.

### 3.4. NFE2

Nuclear factor erythroid 2 (NFE2) is induced by GATA1 and promotes proplatelet formation by upregulating platelet genes, including tubulin β-1 chain (*TUBB1*) [84], *RAB27b* [85], caspase 12 (*CASP12*) [130], and 3-beta-hydroxysteroid dehydrogenase (*HSD3B1*) [131]. Moreover, decreased levels of circulating platelets and increased number of MKs in the bone marrow were observed in *Nfe2* knockout mice [132,133]. However, the expanded MKs were impaired in terms of their aberrant granules, demarcation membrane, and lack of binding activity to fibrinogen [134]. In contrast, ectopic expression of NFE2 in bone marrow cells were found to enhance MK differentiation and platelet production, indicating that NFE2 is crucial for platelet biogenesis [135]. Interestingly, patients with myeloproliferative neoplasms showed upregulation of NFE2 regardless of the presence of the JAK2V617F mutation, presumably due to the increase in AML1 levels [86,136].

### 3.5. MECOM

Heterozygous mutations in *MECOM* (*MDS1* and *EVI1* complex locus) can cause congenital hypomegakaryocytic thrombocytopenia associated with inherited bone marrow failure syndromes [87,88]. Recently, a severe reduction in early CD34^+^CD38^lo^ hematopoietic progenitors in the bone marrow and thrombocytopenia was reported in 12 patients with *MECOM* mutations [89]. In addition, the plasma levels of TPO were elevated although the TPO receptor MPL, which is known to be regulated by EVI1, was detected.

### 3.6. ETV6

The ETS variant 6 gene (ETV6), a member of the ETS family, has been demonstrated to play a role in megakaryopoiesis through transcriptional repression [137]. ETV6 is important for early hematopoiesis in the bone marrow; however, it has little effect on committed lymphoid progenitors. Interestingly, a drastic reduction in platelet counts was observed in MkP-specific knockout of *Etv6*, suggesting that ETV6 is involved in megakaryopoiesis [138]. Accordingly, germline mutations in *ETV6* are related to familial thrombocytopenia (usually mild) and leukemia predisposition [73,90,91]. In addition, although ETV6 is a transcriptional repressor, it has been reported to interact with FLI1 [139].

Immature megakaryocyte hyperplasia and dyserythropoiesis were observed in the bone marrow of patients with the p.P214L mutation [90]. Erythroid transcripts were increased in the platelets of patients with *ETV6* mutation, whereas the platelet transcripts were decreased. This indicates that ETV6 plays a lineage-specific role, by functioning as a transcriptional repressor in erythropoiesis and as a transcriptional activator in megakaryopoiesis [92].

### 3.7. GFI1B

*GFI1B*-related thrombocytopenia (*GFI1B*-RT) is a rare dominant congenital platelet disorder caused by mutations in the *GFI1B* gene. *GFI1B*-RT is characterized by the presence of truncated proteins lacking the zinc fingers of GFI1B, and mild to moderate bleeding disorder with macrothrombocytopenia. Platelets often show alpha granule deficiency, while anisocytosis and poikilocytosis are also observed [93,140]. The gene encodes two protein isoforms: GFI1B-p37 (isoform 1) and GFI1B-p32 (isoform 2). The long isoform 1 is required for megakaryocytic differentiation, while the shorter isoform induces proper erythropoiesis [94,141,142]. Mouse models with knockout or conditional knockout of *Gfi1b* revealed a severe phenotype [143], and thus, a novel *Gfi1b* dominant-negative mouse model was developed to obtain phenotypes similar to those found in humans [95]. Notably, patients with *GFI1B*-RT show hyperplasia of the MkPs and MKs in their bone marrow, which could also be observed in the mouse model. It was demonstrated that treatment with the TPO analog romiplostim, was able to rescue the disease phenotype in the mouse model.

### 3.8. IKZF5

The Ikaros family of transcription factor genes, *IKZF1* through *5*, is widely expressed in hematopoietic cells, and *IKZF1*, *2*, and *3* play roles in lymphocyte development [144]. The immunomodulatory drugs lenalidomide and pomalidomide, were shown to induce the expansion of early megakaryocytic progenitors and thrombocytopenia in patients with multiple myeloma via suppression of *IKZF1* [145]. This leads to the subsequent downregulation of GATA1, as it binds to IKZF1, which further decreases the expression of megakaryocyte genes, including *ZFPM1* and *NFE2* [146].

Recently, it has been found that the rare missense variants of *IKZF5* are associated with thrombocytopenia [96]. The authors performed RNA sequencing of platelets, monocytes, neutrophils, and CD4+ T cells, and the results revealed that the platelets showed dramatic differences in their gene expression. The most significantly downregulated genes, such as *FERMT3, PLA2G4A, P2RY12, TBXA2R, CDC42, GP1BA*, and *GP9*, were related to platelet activation, aggregation, and platelet formation. About half of the differentially expressed genes (DEGs) that were upregulated are involved in immune system response and inflammation. *GATA1* was found to be downregulated, which is consistent with its role in thrombopoiesis [147]. Among the variants of IKZF5, missense mutations of highly conserved residues in the N-terminal zinc fingers exhibited a strong reduction in chromatin binding and inhibited nuclear translocation, suggesting that the mechanism underlies the transcriptional activation of *IKZF5* for platelet formation.

## 4. Clinical Implications of MK Hyperplasia

### 4.1. Thrombocytosis and/or Platelet Activation with Increased Thrombosis Risk

#### 4.1.1. Clonal Hematopoiesis and Thrombocytosis

Clonal hematopoiesis (CH) can be observed in 10–20% of individuals older than 70 years [148,149]. The hematopoietic cells harbor mutations in the epigenetic regulators, such as *DNMT3A* and *TET2*, and CH is considered to increase risks of the developing hematological malignancies as well as cardiovascular diseases [150,151]. Although multiple factors involve in onset of the diseases, increased megakaryopoiesis and thrombopoiesis may be responsible for the CH-enhanced thrombosis risk and cardiovascular symptoms [152]. Among the CH mutations, genes encoding Additional sex combs like 1 (*ASXL1)* [153], Splicing factor 3B subunit 1 *(SF3B1)* [154], DNA methyltransferase 3a *(DNMT3A)* [155], Src homology 2 B3 (*SH2B3*, also named *LNK*) [156], and *JAK2* [157] are considered to be associated with thrombocytosis and/or platelet hyper-reactivity.

Somatic mutations of *ASXL1* are frequently found in hematological malignancies, such as acute myeloid leukemia (AML), chronic myelomonocytic leukemia (CML), and MPN [153]. ASXL1 acts as tumor suppressor in normal hematopoiesis. It has been demonstrated that transgenic expression of mutant *Asxl1* could lead to thrombocytosis, age-related anemia, and myeloid dysplasia [153]. Among genes related to clonal hematopoiesis and predisposition to leukemia, loss-of-function mutation in gene encoding *DNMT3A* is one of the most prevalent. It plays a key role for DNA methylation and tumor suppression in hematopoiesis. Although the overall platelet count in AML patients is lower than healthy population, AML patients carrying *DNMT3A* mutations display higher thrombopoietic potential than patients with wild type *DNMT3A*, which could increase risk of thrombosis [155].

The gain-of-function mutations in *JAK2*, i.e., mutation V617F, are often detected in patients with MPN, which includes polycythemia vera (PV), essential thrombocythemia (ET), and primary myelofibrosis (PMF). JAK2 is a nonreceptor tyrosine kinase, and regulates cell survival and proliferation via cytokine receptor signaling. It has been reported that platelets from *JAK2* V617F-positive patients displayed enhanced procoagulation activity [158]. The *JAK2* V617F also promotes megakaryopoiesis, as JAK2 is critical in signal transduction of thrombopoietin [159]. Notably, the prevalence of mutations in *ASXL1* or the *JAK2* V617F variant is low in ageing healthy individuals; however, the risk of developing CVD has been estimated to twice or 10-fold increase, respectively [157].

#### 4.1.2. Megakaryocyte Hyperplasia in ET and Thrombocytosis

The diagnostic criteria for ET have been announced to be platelet count ≥450 × 10^9^/L, a proliferative bone marrow appearance with megakaryocyte predominance, not meeting the criteria for other myeloid neoplasms, and presence of a *JAK2*, *CALR*, or *MPL* mutation, by the World Health Organization (WHO) [152]. Approximately 55% of *JAK2*-V617F, 15–24% of *CALR*, and 4% of *MPL* mutations are found in ET. It has been suggested that *JAK2* mutation after the first acquisition of *DNMT3A* mutation would lead to an ET phenotype [155]. Patients with ET may experience thrombosis, cardiovascular symptoms, or hemorrhage. Platelet-derived microvesicles (MVs) or microparticles (MPs) have been found to be elevated in peripheral blood of the patients [160]. Importantly, platelet-derived MV/MPs showed procoagulant and prothrombotic activities, which indicate that the risk of thrombosis in ET is correlated with the amount of platelet-derived MV/MPs [161]. Thus, it has been suggested that measurement of the MV/MPs for diagnosis of MPN, ET, or the thrombosis risk of ET might be feasible via liquid biopsy [162].

Chronic inflammation followed by oxidative stress in ET may be associated with the disease progression [163]. Constitutively active JAK-STAT signaling pathway promotes secretion of pro-inflammatory cytokines such as TNF-α and IL-1β, which could lead to increase of ROS production in ET [164]. Notably, ROS impacts on platelet biogenesis and platelet activation, as described in the Section 2. Moreover, leukemia or fibrosis could occur, presumably related to chronic myeloproliferative disorder and MK hyperplasia in the bone marrow [165]. Similar clinical implication has been reported in patients with hereditary thrombocytosis, who have mutations in *TPO* [166].

#### 4.1.3. Cancer and Thrombocytosis

As described in the Section 2.1, various types of tumor cells are known to directly activate platelets and induce TCIPA to protect themselves. Thus, tumor-associated thrombosis is considered to worsen disease prognosis and survival [167]. It is estimated that approximately 20% of cancer patients suffer from vascular thromboembolism like pulmonary embolism, which indicates a greater risk of venous thrombosis in patients with malignancy [168]. Not only platelet hyper-reactivity, but also increased platelet counts can be associated with the severity of cancers, including breast [169], colorectal [170], and lung cancers [171]. Notably, it has been demonstrated that tumor-derived interleukin-6 promoted thrombopoietin production by the liver in ovarian cancer patients [172]. Efforts have been made to elucidate the underlying mechanisms and to develop novel antiplatelet therapeutics for cancer management. In addition to thrombocytosis risk in cancers, chemotherapy for treating cancers is responsible for severe thrombocytopenia. Recently, it has been demonstrated that damage on platelet mitochondria was induced by chemotherapy, and therefore, maintenance of bone marrow megakaryocytes may be crucial for the recovery [173].

### 4.2. MK Hyperplasia and Reduced Platelet Biogenesis

#### 4.2.1. Ageing and Thrombocytopenia

Thrombocytopenia can occur not only in congenital diseases, but also in conditions that induce a low blood platelet count, such as leukemia, immune system disorder, and surgery. Ageing is another factor that affects platelet count and function. In humans, the platelet count is maintained consistently between the ages of 25 to 60, and may later decrease by approximately 8% [174,175,176]. In a study involving 1058 men and 1363 women aged between 26–82 years, it was shown that platelet aggregation, which is induced by epinephrine, ADP, and collagen, tends to decrease with age [177]. Although the platelets remain intact in the elderly population, there is a high prevalence of morbidity caused by other metabolic diseases or immunologic disorders, which may affect platelet biology. Considering the rapid growth of ageing population worldwide and the limited number of studies on patients over 75 years of age, there is an urgent need to elucidate platelet biogenesis and function in the elderly. Moreover, it has been shown that 129 mRNAs and 15 microRNAs are differentially expressed in platelets in the elderly [178].

Age-dependent changes are often associated with changes in bone marrow MKs. For instance, MK-biased hematopoiesis, which is either due to the transcriptional activity of MK precursors or an increase in metabolic stress in the bone marrow microenvironment, has been observed in old mice [6,179,180]. As previously mentioned, ROS can also affect platelet biogenesis. Moreover, ageing-related oxidative stress is higher in the platelets of elderly population [181]. For instance, the levels of thioredoxin-interacting protein (TXNIP), which hinders the endogenous antioxidant thioredoxin-1, were found to be increased in the platelets of older subjects (mean age 67 years) [182]. Platelets from 18-month-old mice have been shown to upregulate peroxide levels [183], suggesting that oxidative stress is related to the changes in platelet characteristics with ageing. Interestingly, the older MkPs harbor a greater capacity to engraft, expand, and generate platelets than young MkPs, presumably due to the enhanced proliferative potential of MkPs [184]. However, the old MkPs could not induce long-term engraftment of myeloid cells and lacked lymphocyte generation. Furthermore, age-related changes in gene expression levels, including that of *Fli1*, *Gabpa*, *Mpl*, *Pf4*, and *Runx1*, indicate that old MkPs may serve as a reservoir for mutations in clonal hematopoiesis during ageing [185].

#### 4.2.2. Primary Myelofibrosis (PMF)

PMF is classified as a *BCR*-*ABL1*-negative MPN that harbor driver mutations in *JAK2*, *CALR*, or *MPL*, representing clonal hematopoiesis [186]. The median age of diagnosis of PMF ranges from 69 to 79 years, and most patients are over 60 years old [187]. Notably, chronic inflammation and ageing may be implicated in diseases such as MPN [188,189].

The levels of inflammatory cytokines such as tumor necrosis factor α (TNF-α) and interleukin 1β (IL-1β), are elevated in aged mice and elderly humans [190,191]. Recently, scRNA-SEQ analysis revealed that the genes related to inflammation, mitochondrial dysfunction, and oxidative phosphorylation were enriched in the MKs isolated from aged mice (>18 months), compared to that of young mice (2–3 months) [45]. In addition, a high incidence of thrombotic events was observed on stimulating the platelets with increased levels of circulating TNF-α [192], while ageing-associated platelet hyperactivity, thrombosis, and mitochondrial dysfunction was reversed by TNF-α blockade [45]. In addition to platelet activity, there were changes in the MKs, including increased MkP population and ploidy of MKs in the bone marrow, indicating that ageing and inflammation enhanced the potential of megakaryopoiesis.

**Figure 3:**
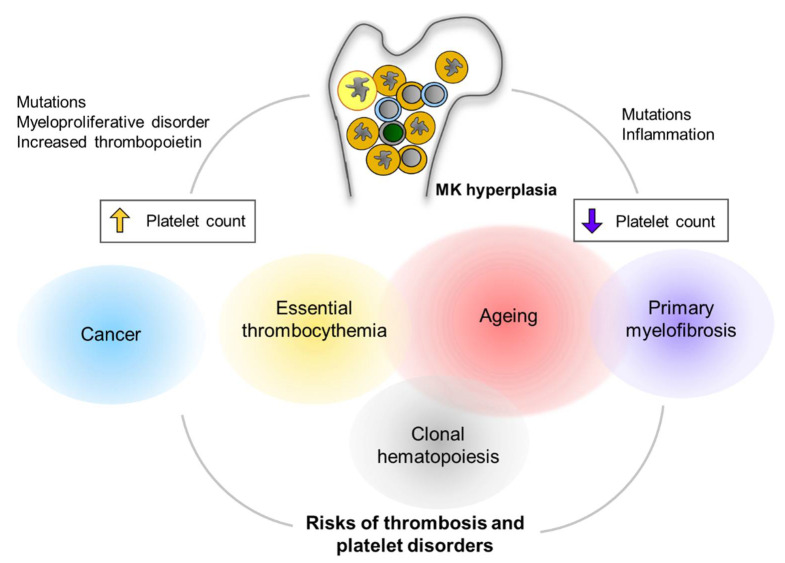
Clinical implications of MK hyperplasia. MK hyperplasia can be seen in many clinical status including clonal hematopoiesis (CH) and myeloproliferative neoplasm (MPN). Mutations in genes that cause CH or MPN are found more frequently in the elderly population. Cancers are another critical risk for thrombocytosis and platelet activation. MK hyperplasia results in either thrombocytosis (increased platelet count) or thrombocytopenia (decreased platelet count), presumably depending on physiological relevance of megakaryopoiesis as well as fibrotic status in the bone marrow. Alterations in platelet number can be implicated to increased risks of thrombus formation and/or platelet disorders.

In both mice and humans, PMF is characterized by increased levels of TNF-α along with an increase in the incidence of thrombosis, caused by platelet hyperactivity [193]. Notably, patients with PMF show MK-biased hematopoiesis, resulting in the accumulation of immature MKs in the bone marrow, extramedullary hematopoiesis, and fibrosis. The number of mitochondria per platelet was also increased in patients with *JAK2* V617F MPN [194], which might be because of decreased autophagy, similar to that seen in the platelets of aged mice [195]. An overview of clinical implications of MK hyperplasia is depicted in Figure 3.

## 5. Conclusions

In this review, I describe the characteristics of platelets and megakaryocytes to understand the mechanism of platelet biogenesis. Although several key regulators of the process, including *RUNX1*, *GATA1*, have been identified, the effects of metabolic changes, microRNAs, and epigenetic regulation remain to be elucidated. Moreover, since MKs are very rare and reside in the bone marrow, there is a lack of proper sources of human MKs for further investigations. Thus, recent advancements in scRNA-SEQ technology have provided valuable information, which I also introduce in the current review. Moreover, emerging evidence suggests that ageing can affect megakaryopoiesis as well as platelet function. Although it is quite clear that chronic inflammation can affect the MKs and platelet biology, the importance of transcription factors, such as their physiological levels and interactions with other factors, have not been elucidated. In addition, although certain diseases are characterized by the occurrence of thrombocytopenia, the bone marrow of the affected patients shows hyperplasia of MKs and MkPs. Taken together, it can be inferred that transcription factors induce megakaryopoiesis, and that they may be associated with many diseases, including thrombocytopenia, metabolic diseases, and MPN.

## Figures and Tables

**Table 1:** Key transcription factors for platelet biogenesis and mutations found in inherited platelet disorders.

Transcription Factor	Target Genes	Alterations of Megakaryopoiesis	Defects in Platelet Function	Disease	Other Features	Ref
RUNX1	PF4, NR4A3, PRKCQ, MYL9	Reduced polyploidization and proplatelet formation	Platelet granule deficiency	Familial platelet disorder with a predisposition to myeloid malignancy (FPDMM)	High risk (>40%) of acute myeloblastic leukemia or MDS at a young age, Absent to moderate bleeding tendency	[76,77,78]
GATA1	NFE2, ITGA2B, Erythropoietic genes (ALAS2, BCL2L1, etc.)	Dysplasia of immature MK and MEP	Platelet alpha granule deficiency, Macrothrombocytopenia, Impaired platelet aggregation	GATA1-related disorders	Dyserythropoiesis with or without anemia, Risk of TMD, DS-AMKL	[79,80,81]
FLI1	MPL, ITGA2B, PF4, GP9	Dysmegakaryopoiesis, Reduced MK production from the patient-derived iPSCs	Macrothrombocytopenia with giant fused alpha granules, Defective platelet aggregation	Paris-Trousseau syndrome (PTS), FLI1-related thrombocytopenia, Jacobsen syndrome	Risk of bleeding	[82,83]
NFE2	TUBB1, RAB27b, CASP12, HSD3B1	Hyperplasia of immature MKs, Impaired DMS in MKs, Lack of binding activity to fibrinogen	Decreased levels of circulating platelets in *Nfe2-*null mice	Patients with MPNs show upregulation of NFE2 regardless of the presence of *JAK2* mutation	Anemia with compensatory reticulocytosis and splenomegaly in *Nfe2*-null mice	[84,85,86]
MECOM	MPL	Hypomegakaryopoiesis	Severe bleeding tendency	*MECOM*-related thrombocytopenia	Bone marrow failure, Elevated TPO levels in plasma, Anemia, B-cell deficiency	[87,88,89]
ETV6	GP1BA, GPIX	Hyperplasia of immature MKs, Dyserythropoiesis, Increased number of circulating HSPCs	Elongated alpha granules in platelets, Impaired adhesion, spreading and clot retraction activity	ETV6-related thrombocytopenia	Leukemia predisposition, Platelets with high levels of transcripts for erythrocytes, Absent to mild bleeding	[90,91,92]
GFI1B	RGS18	Hyperplasia of MK and MkPs	Macrothrombocytopenia, Alpha granule deficiency, Defects in platelet aggregation, Reduced expression of GP1ba	*GFI1B*-related thrombocytopenia (*GFI1B*-RT)	Anisocytosis and poikilocytosis of RBCs, Mild to severe bleeding, Dyserythropoiesis, Severe phenotypes in mice model	[93,94,95]
IKZF5	Platelet activation genes (LYN, P2RY12, etc.)	N/A	Platelets with reduced alpha and delta granules	*IKZF5*-related thrombocytopenia	Downregulation of gene expression for platelet biogenesis including *GATA1*, No bleeding tendency	[96]

*RUNX1,* Runt-related transcription factor 1; *PF4,* Platelet Factor 4; *NR4A3,* Nuclear Receptor subfamily 4 group A member 3; *PRKCQ,* Protein Kinase C theta; *MYL9,* Myosin Light Chain 9; *MDS,* Myelodysplastic Syndrome; *NFE2,* Nuclear Factor Erythroid 2; *ITGA2B,* Integrin αIIb; *ALAS2,* 5′-Aminolevulinate Synthase 2; *BCL2L1,* BCL2 Like 1; *MEP,* Megakaryocyte-Erythroid Progenitor; *TMD,* Transient Myeloproliferative Disorder; *DS-AMKL,* Down Syndrome Acute Megakaryoblastic Leukemia; *FLI1,* Friend Leukemia virus Integration 1; *MPL*, thrombopoietin receptor; *iPSC,* induced Pluripotent Stem Cell; *TUBB1,* Tubulin Beta 1; *CASP12,* Caspase 12; *HSD3B1,* 3-beta-Hydroxysteroid Dehydrogenase; *DMS,* Demarcation System; *MECOM,* MDS1 and EVI1 Complex Locus; *ETV6,* ETS Variant 6; *HSPC*, Hematopoietic Stem and Progenitor Cell; *RGS18*, Regulator of G protein Signaling 18; *RBC,* Red Blood Cell; *P2RY12,* P2Y Purinoceptor 12; *N/A*, Not Applicable.
